# Biofilm Formation and Antibiotic Resistance Phenotype of *Helicobacter pylori* Clinical Isolates

**DOI:** 10.3390/toxins12080473

**Published:** 2020-07-24

**Authors:** Kartika Afrida Fauzia, Muhammad Miftahussurur, Ari Fahrial Syam, Langgeng Agung Waskito, Dalla Doohan, Yudith Annisa Ayu Rezkitha, Takashi Matsumoto, Vo Phuoc Tuan, Junko Akada, Hideo Yonezawa, Shigeru Kamiya, Yoshio Yamaoka

**Affiliations:** 1 Department of Environmental and Preventive Medicine, Oita University Faculty of Medicine, Yufu 879-5593, Japan; kartikafauzia@gmail.com (K.A.F.); doctordoohan@gmail.com (D.D.); tmatsumoto9@oita-u.ac.jp (T.M.); vophuoctuandr@gmail.com (V.P.T.); akadajk@oita-u.ac.jp (J.A.); 2 Institute of Tropical Disease, Universitas Airlangga, Surabaya 60115, Indonesia; langgengaw@gmail.com (L.A.W.); yudithannisaayu@gmail.com (Y.A.A.R.); 3 Division of Gastroentero-Hepatology, Department of Internal Medicine, Faculty of Medicine-Dr. Soetomo Teaching Hospital, Universitas Airlangga, Surabaya 60115, Indonesia; 4 Division of Gastroenterology, Department of Internal Medicine, Faculty of Medicine, University of Indonesia, Jakarta 10430, Indonesia; ari_syam@hotmail.com; 5 Department of Internal Medicine, Faculty of Medicine, Universitas Muhammadiyah Surabaya, Surabaya 60113, Indonesia; 6 Department of Infectious Diseases, Kyorin University School of Medicine, 6-20-2 Shinkawa, Mitaka, Tokyo 181-8611, Japan; yonezawa@ks.kyorin-u.ac.jp (H.Y.); skamiya@ks.kyorin-u.ac.jp (S.K.); 7 Department of Medicine, Gastroenterology and Hepatology Section, Baylor College of Medicine, Houston, TX 77030, USA; 8 Global Oita Medical Advanced Research Center for Health (GO-MARCH), Yufu 879-5593, Japan; 9 Borneo Medical and Health Research Centre, University Malaysia Sabah, Kota Kinabalu, Sabah 88400, Malaysia

**Keywords:** *Helicobacter pylori*, antibiotic resistance, biofilm formation, biofilm-specific resistance, amoxicillin, clarithromycin, levofloxacin, metronidazole, tetracycline

## Abstract

We evaluated biofilm formation of clinical *Helicobacter pylori* isolates from Indonesia and its relation to antibiotic resistance. We determined the minimum inhibition concentration (MIC) of amoxicillin, clarithromycin, levofloxacin, metronidazole and tetracycline by the Etest to measure the planktonic susceptibility of 101 *H. pylori* strains. Biofilms were quantified by the crystal violet method. The minimum biofilm eradication concentration (MBEC) was obtained by measuring the survival of bacteria in a biofilm after exposure to antibiotics. The majority of the strains formed a biofilm (93.1% (94/101)), including weak (75.5%) and strong (24.5%) biofilm-formers. Planktonic resistant and sensitive strains produced relatively equal amounts of biofilms. The resistance proportion, shown by the MBEC measurement, was higher in the strong biofilm group for all antibiotics compared to the weak biofilm group, especially for clarithromycin (*p =* 0.002). Several cases showed sensitivity by the MIC measurement, but resistance according to the MBEC measurements (amoxicillin, 47.6%; tetracycline, 57.1%; clarithromycin, 19.0%; levofloxacin, 38.1%; and metronidazole 38.1%). Thus, biofilm formation may increase the survival of *H. pylori* and its resistance to antibiotics. Biofilm-related antibiotic resistance should be evaluated with antibiotic susceptibility.

## 1. Introduction

A decrease in the cure rate of *Helicobacter pylori* infection after treatment with the first-line regimen has been reported in various areas of the world as a result of high antibiotic resistance [1]. Persistent infection with *H. pylori* can induce the development of gastroduodenal diseases such as peptic ulcer disease and gastric cancer. In an attempt to solve this problem, a recent consensus on *H. pylori* treatment strongly recommended tailoring treatment guided by antibiotic susceptibility testing results [2]. It aimed to increase the success rate and decrease the antibiotic resistance status worldwide, but eradication failures were still reported in several studies [3,4]. These data suggested that there was a gap in clarifying the mechanism of antibiotic resistance. Understanding the mechanisms of antibiotic resistance can contribute to effective eradication and control of *H. pylori* infection.

In addition to the resistance mechanism, due to mutations on targeted genes conferring antibiotic resistance, biofilm formation appears to have become another important factor affecting susceptibility [5]. A biofilm consists of an exopolysaccharide matrix layer that covers sessile bacteria [6]. In contrast, free-swimming solitary bacteria (planktonic) have a metabolic state and transcription property different from that of bacteria in a biofilm [7]. The biofilm plays an essential role in the survival against external threats and toxic materials including antimicrobial drugs [8]. *H. pylori* can also form a biofilm, which was initially observed covering the gastric surface in an infected patient with a gastric ulcers [9,10]. Our previous study on *H. pylori* described the role of the biofilm in decreasing the susceptibility of a very strong biofilm former to clarithromycin and proved that its biofilm formation might increase mutation rate and resistance [11]. However, the study was limited to a few strains. Moreover, a variation in the amount of biofilm production was reported from weak to strong biofilm-formers [12]. Therefore, the role of the biofilm in antibiotic resistance, especially in clinical strains, remains elusive.

The main purpose of the antibiotic susceptibility test is to provide enough supporting data for choosing a treatment regimen. Currently, the antibiotic susceptibility test used is based on the Clinical and Laboratory Standard Institute (CLSI) guidelines and measures the minimum inhibitory concentration (MIC) in the planktonic stage [13]. However, the biofilm has been proposed to increase resistance in distinct ways from planktonic resistance [14]. Hence, the MIC measurement may not be able to detect resistance in the biofilm state [15,16], and this may affect the treatment outcome [7,17,18]. Thus, biofilm-associated resistance should be taken into account when determining susceptibility. Another tool to evaluate biofilm susceptibility, the minimum biofilm eradication concentration (MBEC), helps to elucidate the role of biofilm in bacterial survival after antibiotic treatment [17,19].

In this study, we aimed to evaluate biofilm formation of clinical isolates obtained from an Indonesian nationwide survey. Indonesia is a country with high prevalence of levofloxacin (31.2%) and metronidazole (46.7%); low prevalence of amoxicillin (5.2%), tetracycline (2.6%) and clarithromycin (9.1%) [20]. Theoretically, standard triple therapy could be used (less than 15% resistant of clarithromycin) [2]. However, a recent clinical trial in Indonesia using the standard triple therapy for 10 days only obtained a 67.6% cure rate of intention to treat (ITT) [21]. This value is much lower than the ideal ITT for the regimen; 90–95% [22,23]. Hence, elucidating the role of biofilm formation in Indonesia is urgently needed. We also investigated the correlation between planktonic resistance, biofilm formation and biofilm resistance to five commonly used antibiotics: amoxicillin, clarithromycin, metronidazole, levofloxacin and tetracycline.

## 2. Results

### 2.1. Distribution of Biofilm Formation

A total of 101 Indonesian strains were analyzed in this study. Those isolates were collected from 66 male and 35 female patients. Among the patients, 90.1% (91/101) were diagnosed with gastritis, 8.9% (9/101) with peptic ulcer disease and one (1.0%; 1/101) had gastric cancer. Even though there was no significant difference in biofilm formation between diseases, strong biofilm formation was observed with the strain MER20 isolated from a gastric cancer patient from Papua Province, Indonesia. Further study with a larger number of gastric cancer cases should be necessary to confirm the association of biofilm in cancer pathogenesis.

**Figure 1:**
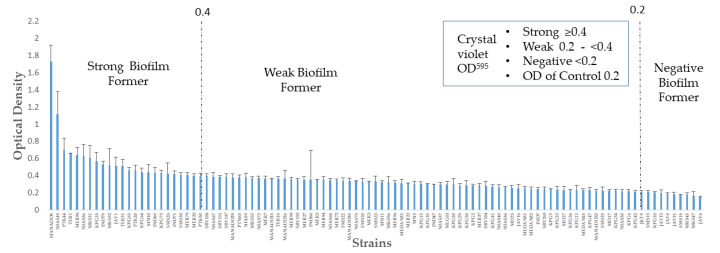
Distribution of biofilm formation in 101 Indonesian patients. The X-axis represents the *H. pylori* strain and the Y-axis represents the optical density from the crystal violet assay to determine biofilm formation among the strains. Based on the criteria above, the strains were differentiated into strong, weak and negative biofilm-formers.

Biofilm formation quantification by crystal violet was performed on the isolates, and the results are summarized in Figure 1. The results yielded OD_595_ values of the 101 strains from 0.149 ± 0.011 (minimum) to 1.732 ± 0.187 (maximum) with a median value of 0.329. The OD_595_ value of the negative control was 0.2 and was set as the cut-off for the biofilm-forming strain. Accordingly, 93.1% (94/101) of the strains showed an OD more than 0.2 and were defined as biofilm-formers. The strains were divided into three groups, negative (less than the 0.2 cut-off), weak (0.2–0.4) and strong (greater than or equal to 0.4) biofilm-formers. Seven (6.9%) isolates showed biofilm OD below 0.2, defined as negative biofilm formers. Among the biofilm-formers, weak biofilm-formers (75.5%, 71/94) were predominant, while strong biofilm formers were 24.5% (23/94).

### 2.2. Biofilm Formation Level among Planktonic Sensitive and Resistant Isolates

We checked the planktonic susceptibility of all strains and analyzed the MIC. Among 101 strains, the highest resistance rate was observed for metronidazole; 49.0% (50/101), followed by levofloxacin; 35.6% (36/101). The resistance rate was low for clarithromycin [7.9% (8/101)], amoxicillin [3.9% (4/101) and tetracycline [3.0% (3/101)].

**Figure 2:**
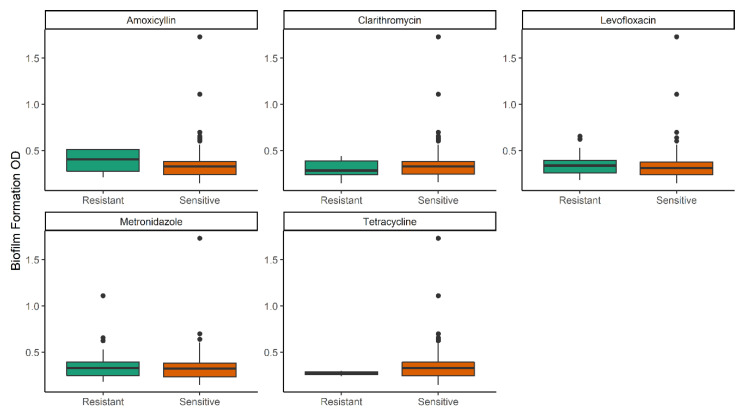
Biofilm formation in the planktonic sensitive and resistant strains. All outliers for amoxicillin, clarithromycin and tetracycline were found in the sensitive strains. There were no significant differences between planktonic sensitive and resistant groups. The *p*-values obtained by the Mann-Whitney analysis were as follows: amoxicillin (*p* = 0.59), clarithromycin (*p* = 0.56), levofloxacin (*p* < 0.43), metronidazole (*p* = 0.74) and tetracycline (*p* = 0.29).

We then compared the biofilm formation level (OD_595_) between the planktonic-sensitive and resistant strains (Figure 2). There were no significant differences between the two groups even though several outliers were observed in the sensitive group for all antibiotics. Hence, these results showed that the biofilm formation level was the same between the planktonic sensitive and resistant isolates. Concordant with this result, the median of MICs among strong, weak and negative biofilm-formers were similar as well (Table S1).

### 2.3. Biofilm Formation and Planktonic Multidrug Resistance

We evaluated the relationship between biofilm formation and the multidrug-resistance pattern, which was defined as planktonic resistance to three or more antibiotics (Table 1). There was no significant difference (*p* = 0.40 by the Kruskal-Wallis test) in the median of the biofilm OD among drug resistance pattern; even though all multidrug-resistant isolates were weak biofilm-formers (Table 1). There was no association between the resistance patterns and biofilm group (biofilm producer vs. negative; *p* = 0.68).

### 2.4. Antibiotic Resistance in the Biofilm Form and Planktonic Form

**Figure 3:**
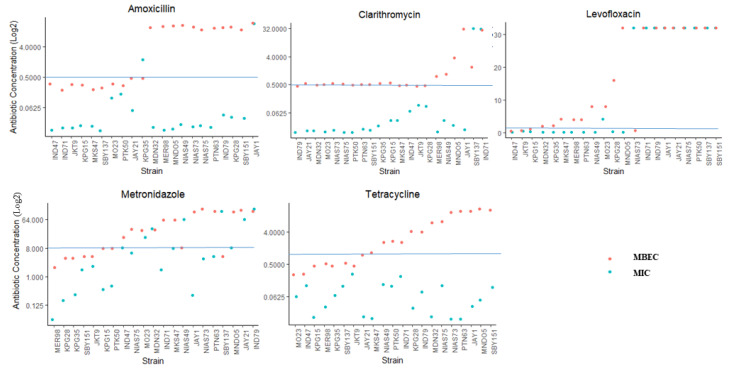
The value of the minimum biofilm eradication concentration (MBEC) is higher than the minimum inhibition concentration (MIC). The x-axis shows the strains arranged from the lowest (left) to the highest (right) MBEC. The y-axis is the log2 conversion scale of the MIC (mg/L) and MBEC (mg/L). The blue line represents the clinical breakpoint for each of the antibiotics. MIC and MBEC values were significantly different (*p* < 0.001) in all antibiotics, as shown by Mann-Whitney analysis, except for levofloxacin (*p* = 0.13).

In contrast with the MIC measurement, the MBEC represents the susceptibility of bacterial cells in the biofilm to antibiotics. Hence, we performed MBEC analysis on 21 isolates by stratified sampling of strong, weak and negative biofilm-forming strains. The measured MBEC values were generally higher than the MIC values (Figure 3). There was a significant difference between MIC and MBEC for all antibiotics (*p* < 0.001), except for levofloxacin (*p* = 0.13).

In amoxicillin, a 1000-fold difference between MBEC and MIC was observed with median MIC values of 16 mg/L and 0.016 mg/L, respectively. For clarithromycin and tetracycline, the MBEC was 31.25-fold higher than the MIC. The median MBEC was 0.5 mg/L and 2 mg/L, while the median MIC was 0.016 mg/L and 0.064 mg/L for clarithromycin and tetracycline, respectively. Meanwhile, in the case of levofloxacin and metronidazole, the median MBEC was 16-fold and 8-fold higher, respectively, than the median MIC.

The samples were then classified as sensitive or resistant with a clinical breakpoint of >0.5 mg/L for amoxicillin [24] and clarithromycin; >1 mg/L for levofloxacin and tetracycline and >8 mg/L for metronidazole (shown in Figure 3 as the horizontal line). In these 21 isolates, we analyzed the resistance proportion measured by the MIC and MBEC. Different proportions of MIC resistance were observed from 0.0% in tetracycline as the lowest to 47.6% (10/21) as the highest in levofloxacin (Table 2). In general, the MBEC resistant proportion was higher than the MIC resistant proportion in case of all antibiotics; this difference was statistically significant in case of amoxicillin and tetracycline, with *p* = 0.006 and *p* < 0.001, respectively. Further analysis showed that the proportion of MBEC resistant organisms (resistant to clarithromycin) was significantly higher in the strong biofilm-forming group than in the weak and negative biofilm-forming groups (83.3%, 5/6 vs. 6.7%, 1/15, *p* = 0.002).

Because of the difference between MIC and MBEC measurements, there were many cases that showed sensitivity by the MIC measurement but resistance by the MBEC measurement; 47.6% (10/21) for amoxicillin, 57.1% (12/21) for tetracycline, 19.0% (4/21) for clarithromycin, 38.1% (8/21) for levofloxacin and 38.1%(8/21) for metronidazole (Table S2 and Figure 3). All isolates that showed resistance via the MIC also showed resistance based on the MBEC to amoxicillin, tetracycline and clarithromycin. Only one case in levofloxacin and two cases in metronidazole were MIC-resistant but were MBEC-sensitive.

### 2.5. The Correlation between Biofilm Amount and Antibiotic Resistance in Biofilm-Forming Groups

**Figure 4:**
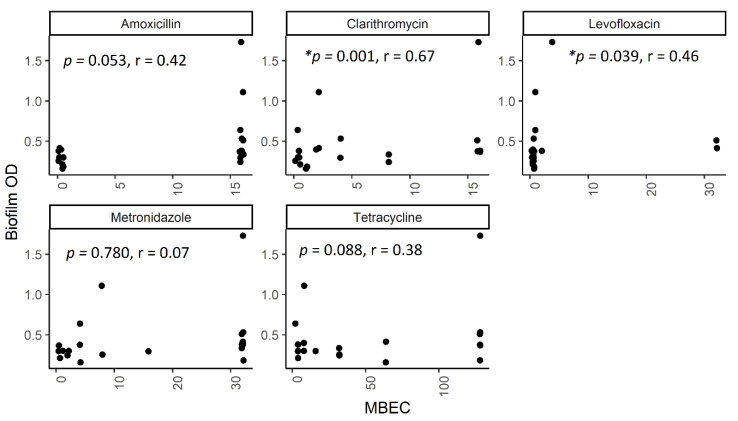
Scatter plot of correlation between MBEC and biofilm OD. The plot represents positive correlation between the MBEC (mg/L) and biofilm formation on 595 nm optical density.

To understand the role of biofilm density in biofilm resistance, we analyzed the correlation between biofilm OD and MBEC data of 21 strains (Figure 4). The spearman rank correlation analysis showed a significant correlation between the biofilm OD and the MBEC results in clarithromycin (*r* = 0.672 and *p* < 0.001) and levofloxacin (*r* = 0.455 and *p* = 0.04). This suggests a positive correlation between biofilm density and the MBEC.

### 2.6. The Effect of Antibiotic Exposure on Biofilm Formation

In biofilm formation screening, an outlier strain, MANADO5, with a very robust biofilm was observed. For confirmation, scanning electric microscope (SEM) analysis was performed. We also evaluated the biofilm biomass of the strain after 24-h exposure to antibiotics at a concentration two-times that of the clinical breakpoint. The biofilm biomass in all amoxicillin, levofloxacin, tetracycline, and metronidazole groups was decreased, but the results were not statistically significant (Figure S1). Biofilm morphology was also observed with SEM, and it showed that for all antibiotics-treated biofilms there was no significant difference in shape, attachment or density between the strains at 24 h (Figure S2).

## 3. Discussion

*H. pylori* growth in a biofilm has been proposed to increase antibiotic resistance in many bacteria [7]. Here, we investigated biofilm formation and resistance phenotype in clinical isolates. The ability of *H. pylori* to produce biofilm was assessed by the in vitro biofilm formation test. Among 101 clinical isolates of *H. pylori,* the majority (93.1%) of the strains collected in the national surveys in Indonesia could produce a strong or weak biofilm. The variation in strong and weak biofilms from a number of clinical isolates was also previously reported [12]. Hence, biofilm formation in the majority of *H. pylori* strains should affect antibiotic resistance in the human stomach.

In addition to performing planktonic antibiotic susceptibility tests to determine MIC values, the MBEC analysis, which assesses the viability of the bacteria after exposure to antibiotics taking into account the biofilm, was also performed [17]. Previous *H. pylori* biofilm studies analyzed the laboratory-adapted strain 26695 [25,26] or a smaller number of clinical isolates (only 1 to 3 strains) [11,27,28]. Those studies reported that the biofilm former cells were more resistant to antibiotics. We therefore attempted to bridge those findings to a large set of clinical isolates that were proportionally defined according to biofilm level. Consistent with previous studies in *H. pylori,* the results showed significantly higher MBEC values compared to MIC values for amoxicillin, clarithromycin, tetracycline and metronidazole. This result suggests that biofilm might protect bacteria from the antibiotic effect, as also reported for bacteria other than *H. pylori* [19,27,28,29]. When we compared susceptibility according to the MIC and MBEC measurements, we observed a higher resistance rate in MBEC, especially to amoxicillin and tetracycline. Attention should be given to these results because a study reported that the high resistance rate shown by the MBEC was related to treatment failure in 81% of *Staphylococcus aureus* infections in peritoneal dialysis [18]. Moreover, amoxicillin is one of the core antibiotics for first-line empirical therapy for *H. pylori* infection [2,24]. Therefore, biofilm-related resistance should be considered as one cause of treatment failure. For clarithromycin, we also observed that the resistant proportion determined by MBEC measurement was significantly higher in the strong biofilm-forming group than in the weak biofilm-forming group. Correlation analysis between biofilm optical density and the MBEC measurement also showed a strong positive correlation. These results indicated that higher biofilm density might increase biofilm resistance.

Even though the MBEC was higher than the MIC among the majority of the strains, several strains showed that the MIC was higher than the MBEC. These strains might gain or lose some mutations during the experiments [30]. Our results also showed that the correlation between the antibiotics and the strength of the biofilm was different among the antibiotics. This variation was probably due to the involvement of multiple factors [7,31,32]. It has been reported in other bacteria that first, several layers of exopolysaccharide and protein in the biofilm that can inhibit antibiotic penetration caused resistance to antibiotics [33]. Second, after exposure to the antibiotic, each colony could repopulate independently after treatment discontinuation [29]. Third, biofilm also triggers bacteria to be in a slow-growth phase that reduces susceptibility to antibiotics [34,35], which is also known as ‘antibiotic tolerance’ [14]. These possible mechanisms should be confirmed in *H. pylori* in a future study. These mechanisms were supported by the decrease in metabolite of the biofilm form of *H. pylori* compared to the planktonic strain [36]. An increasing efflux pump was also observed in biofilm-growing *H. pylori* [27,28].

The association of a stronger biofilm with the planktonic resistant strain and the multidrug-resistant strain has been shown in other biofilm-forming bacteria such as *Pseudomonas aeruginosa* [37]. The inverse correlation was observed in other bacteria such as *Acinetobacter baumannii*, *Stenotrophomonas maltophilia*, and members from *Enterobacteriaceae;* the strong biofilm-formers were likely observed in sensitive strains [19,38,39]. The tendencies allow the clinicians to expect the possible involvement of biofilm in the planktonic susceptibility test. However, the result in our study revealed that the planktonic susceptibility measurement was less likely to predict biofilm role in resistance. The biofilm formation levels in the planktonic sensitive and resistant groups were equal. Similar biofilm formation was also observed between planktonic single-, double-, and multidrug-resistant groups. Therefore, the assessment of biofilm formation and/or biofilm-related resistance (such as by MBEC) should be performed in *H. pylori*. This measurement has been recommended in staphylococcal infection [16]. In a cystic fibrosis case caused by biofilm former *P. aeruginosa,* the treatment guided by the biofilm susceptibility test was more likely to succeed than the treatment based on the planktonic susceptibility test alone [40]. Biofilm resistance measurement could support the clinician in improving the treatment choice, especially in cases of treatment failure.

We also investigated the effect of antibiotics on biofilm formation. However, it seems that antibiotic exposure to the biofilm could not decrease the biofilm density or morphology changes as observed by SEM observation. This might further increase the biofilm threat level. Once a mature biofilm is formed, an antibiotic might have difficulty modulating or removing the biofilm. This result is concordant with a previous study that mentioned that fluoroquinolone and clarithromycin are unable to modify preformed biofilms [41,42,43,44]. However, the ability of several antibiotics to reduce biofilm is probably strain-specific [45]. A combination of drugs, or increasing concentration, potentially results in a destructive effect, as mentioned in other studies [27,41]. The addition of a substance with an antibiofilm effect could be considered as adjuvant therapy, such as N-acetylcysteine, rhamnolipid, and lipid nanoparticle [46,47,48,49].

This study has several limitations. First, a small number of isolates resistant to clarithromycin, amoxicillin, and tetracycline obtained in this study may affect the statistical test, even though all isolates collected from our nationwide survey were included in the analysis. We performed the endoscopy survey for 1172 subjects. However, since the prevalence of *H. pylori* in Indonesia is very low, only 101 strains could be obtained; although this is the largest *H. pylori* culture stock of Indonesian strains in the world. Second, stratified random sampling according to the biofilm level was aimed to minimalize the error during laborious MBEC analysis while still proportionally analyzing the association between biofilm and resistance; but it could also affect the statistical analysis. The data set may contain many resistant strains that have almost similar values between MIC and MBEC. This probably explains the statistical discrepancy in levofloxacin. Table 2 shows that the MBEC was not significantly different (*p* = 0.28) even though Figure 4 shows a significant association between biofilm formation and MBEC (*p* = 0.04).

From the results in this study, there is a high chance that the biofilm explains the low ITT of the cure rate by standard triple therapy of *H. pylori* in Indonesia, despite the low (planktonic) resistant prevalence of clarithromycin and amoxicillin, though further clinical trials are necessary. Indeed, the biofilm formation quantification and MBEC for *H. pylori* in this study were essential to understand the effect of biofilm on antibiotic resistance. This result could be an important stepping-stone to pave the way for improving the efficacy of eradication by taking account biofilm formation in the susceptibility test.

## 4. Conclusions

This study showed two findings regarding the danger of biofilm formation in antibiotic resistance. First, most *H. pylori* isolates produce different levels of biofilm, and there was an equal frequency of biofilm formation in planktonic sensitive and resistant strains. Second, biofilm formation increased bacterial survival after exposure to antibiotics. Therefore, biofilm-related antibiotic resistance should be evaluated. Further studies are necessary to understand the mechanism underlying antibiotic resistance in *H*. *pylori* and to find an appropriate treatment strategy against *H*. *pylori* infections.

## 5. Materials and Methods

### 5.1. Patient Sampling and H. pylori Isolates

We obtained gastric biopsy specimens from 1,172 dyspeptic patients from 18 cities in 8 different islands in Indonesia (Sumatra, Java, Sulawesi, Kalimantan, Bali, Timor, Ternate, and Irian) as mentioned in our previous studies [20,50,51,52]. Because of the low prevalence of *H. pylori* in Indonesia [53], a total of 106 *H. pylori* strains were successfully isolated. We excluded 5 isolates with inadequate growth, and 101 strains were finally included in this study. All the *H. pylori* isolates were subcultured from the bacterial stock in Brucella broth containing 10% glycerol and 10% horse serum in a −80 °C freezer at the Department of Environmental and Preventive Medicine, Oita University Faculty of Medicine, Japan. All patients from whom the isolates were obtained provided written informed consent, and the protocol was approved by the Ethical Committee of Oita University Faculty of Medicine (Yufu, Japan, P-12-10, 18 January 2013), Dr. Soetomo Teaching Hospital (Surabaya, Indonesia, 221/Panke.KKE/IX/2012, 25 September 2012), and Dr. Cipto Mangunkusumo Teaching Hospital (Jakarta, Indonesia, 206/112/P1/ETIK/2014 7 April 2014).

### 5.2. Antibiotic Susceptibility Test

We determined the MIC by the Etest (Biomerieux, Nice, France) as a measure of the susceptibility of the planktonic form of 101 strains to five antibiotics [54], including 77 strains from our previous study [20]. The isolates were subcultured three times from the −80 °C bacterial stock in a Brucella agar plate supplemented with 7% horse blood without antibiotics in microaerophilic conditions. On day two, *H. pylori* cultures were collected in the Phophate Buffer Saline and adjusted into 3.0 Mc Farland standard. The *H. pylori* cultures were inoculated into Mueller Hinton agar (Becton Dickinson, Le Pont de Claix, France) supplemented with 5% horse blood, and an Etest strip was placed in the middle of the plate. The concentration of clarithromycin, amoxicillin, tetracycline and metronidazole ranged from 0.016 mg/L to 256 mg/L, while levofloxacin ranged from 0.002 mg/L to 32 mg/L, according to the concentration available from the manufacturer. The evaluation was performed after 72 h of incubation in microaerophilic conditions. We used *H. pylori* 26695 as the standard strain. We also determined the MIC50 as the same value of the median to represent the ability of the antibiotic to inhibit ≥50% of strains. The clinical breakpoint of each antibiotic was as follows: amoxicillin, 0.125 mg/L; clarithromycin, 0.5 mg/L; levofloxacin, 1 mg/L; metronidazole, 8 mg/L and tetracycline, 1 mg/L, according to the clinical breakpoint published by the European Committee on Antimicrobial Susceptibility Testing (EUCAST) for the antibiotic susceptibility test [55].

### 5.3. Biofilm Quantification

Biofilm quantification was performed in triplicate using the crystal violet method for *H. pylori* as previously described with modifications [11]. Briefly, the bacteria that grew in the blood plate were collected in 1 mL Brucella broth supplemented with 10% fetal bovine serum (FBS) medium and pre-cultured for 24 h in microaerophilic conditions. The bacterial suspension was adjusted to an OD of 0.4 (approximately 2.5 × 10^6^/mL), and 25 µL of *H. pylori* suspension was inoculated into 24 well plates containing 1 mL medium. These plates were incubated in the microaerophilic environment with shaking (100 rpm) for 3 days. The planktonic cell suspension was then removed. The plates were washed with PBS and air-dried for 1 h to create stronger attachment of the biofilm to the wall. After that, the biofilm was stained with 500 µ L of 0.01% crystal violet for 1 min, washed with distilled water and then air-dried for 15 min. The crystal violet was then diluted with a 500 µL mixture of ethanol and acetic acid (ethanol:acetic acid = 95:5). The quantity of biofilm was obtained from absorbance measurements with a spectrophotometer (Multiskan Go, Thermo Fischer, Yokohama, Japan) at a wavelength of 595 nm. The measurement of the well containing medium without bacteria was used as a negative control, and strain TK1402, that has been reported as strong biofilm former [56], was used for the positive control. The biofilm was classified as negative if the measured OD was lower than the control. Biofilm formation was classified into three groups: a negative biofilm-former had an OD < control OD, a weak biofilm-former had an OD ≥ the control OD, and <2 times of the control OD, and a strong biofilm-former had an OD ≥ 2 times of the control OD [29,57,58,59].

### 5.4. Biofilm Formation and MBEC

The survival of bacteria after antibiotic exposure was measured by MBEC as mentioned in the previous study, with modifications [11,19,28]. Twenty-one representative strains were selected by stratified random sampling according to biofilm formation and we performed the analysis in triplicate. Briefly, we inoculated 25 µL of an *H. pylori* suspension with an OD of 0.4 into 925 µL of Brucella broth and 10% FBS in 24-well plates and let the biofilm grow in the microaerophilic environment at 37 °C with shaking. On day 3, we exposed the bacteria, including those strains that lived under a biofilm, by adding 50 µL serial concentrations of antibiotics for 24 h. After 24-h incubation in a microaerophilic environment, the liquid medium was removed. Then, the attached biofilm on the plate’s wall was manually scraped after the addition of 500 µL PBS. The bacterial suspension (2.5 µL) was then inoculated on a Brucella agar plate supplemented with 7% FBS and cultured under a microaerophilic environment for 3 days. The 21 strains that did not undergo treatment with the antibiotic process were also spotted on the same plate as the control.

### 5.5. Scanning Electron Micrograph Analysis

For SEM analysis, the biofilms were grown on coverslips (18 × 18 mm) in 12-well plates. We added 50 µL of bacterial suspension (OD, 0.4) to 2 mL of *Brucella* broth with 10% FBS, followed by incubation for 4 days with shaking under microaerophilic conditions. To evaluate biofilm formation after exposure with antibiotics, we also grew the biofilm for 3 days and exposed each biofilm to five antibiotics at a concentration two-times higher than that of each clinical breakpoint for 24 h. All the coverslips with biofilms were washed with PBS and fixed with 50% glutaraldehyde, dehydrated with ethanol from a concentration of 50 to 100%, and then washed with 50% t-butyl alcohol and 100% t-butyl alcohol. Following the washing process, the coverslip was exposed to osmium for 10 min, dried, and covered with gold. SEM analysis was performed using a Scanning Electron Microscope (S-4800, Hitachi, Tokyo, Japan).

### 5.6. Statistical Analysis

We examined the effect of biofilm production on the susceptibility of the strain with the Mann Whitney U test and the Chi-square test. The other non-parametric correlation was analyzed by the Spearman rank correlation model. All statistical analyses and graph construction were performed in the R environment (version 3.5.1, R Foundation for Statistical Computing, Vienna, Austria).

## Figures and Tables

**Table 1:** Multidrug-resistance pattern and biofilm formation (total *n* = 101).

Drug-Resistance Pattern	Median Biofilm Formed	*n* (%)	Biofilm Formation *n* (%)		
			Strong	Weak	Negative
All Sensitive	0.314	33 (32.7)	6 (26.1)	23 (32.4)	4 (57.1)
Single-Resistant	0.333	42 (41.6)	9 (39.1)	31 (43.7)	2 (28.5)
Double-Resistant	0.344	21 (20.8)	8 (34.8)	12(16.9)	1 (14.3)
Multidrug-Resistant	0.276	5 (4.9)	0 (0)	5 (7.0)	0 (0)
Total		101	23	71	7

**Table 2:** MIC and MBEC comparison (total *n* = 21).

	Resistance Proportion (%)				
	Amoxicillin	Clarithromycin	Levofloxacin	Metronidazole	Tetracycline
All MIC	2/21 (9.5)	2/21 (9.5)	10/21 (47.6)	6/21 (28.6)	0/21 (0.0)
All MBEC	11/21 (52.4)	6/21 (28.6)	17/21 (80.9)	12/21 (57.1)	12/21 (57.1)
*p* *	0.006	0.24	0.051	0.12	<0.001
MIC					
Weak	1/15 (6.7)	1/15 (6.7)	7/15 (46.7)	4/15 (26.7)	0/15 (0.0)
Strong	1/6 (16.7)	1/6 (16.7)	3/6 (50)	2/6 (33.3)	0/6 (0.0)
*p* **	0.5	0.5	0.99	0.99	-
MBEC					
Weak	6/15 (40.0)	1/15 (6.7)	11/15 (73.3)	8/15 (53.3)	7/15 (46.7)
Strong	5/6 (80.0)	5/6 (83.3)	6/6 (100)	4/6 (66.7)	5/6 (83.3)
*p* ***	0.15	0.002	0.28	0.66	0.18

* *p* was obtained by the Fisher exact test between MIC resistance proportion and MBEC resistance proportion. ** *p* was obtained by the Fisher exact test between MIC resistance proportion among weak and strong biofilm. *** *p* was obtained by the Fisher exact test of MIC resistance between weak and strong biofilm.

## Supplementary Materials

The following are available online at https://www.mdpi.com/2072-6651/12/8/473/s1, Figure S1: The effect of 24-h antibiotic exposure on the mature biofilm mass, Figure S2: The SEM images of the biofilm of strain MANADO5 24 h after antibiotic exposure at a dose 2 times that of the clinical breakpoint, Table S1: Association of planktonic susceptibility and biofilm formation, Table S2: The different proportion of resistance obtained by MIC and MBEC measurement.
