# Supplementary material for: Biofilm Formation and Antibiotic Resistance Phenotype of Helicobacter pylori Clinical Isolates
Source: Toxins (Basel). 2020 Jul 24;12(8):473. doi: 10.3390/toxins12080473 (PMC7472329; doi:10.3390/toxins12080473)
Supplement: Supplementary file 1 [file toxins-12-00473-s001.pdf]

Supplementary Materials: Biofilm Formation and Antibiotic Resistance Phenotype of *Helicobacter pylori* Clinical Isolates

Kartika Afrida Fauzia, Muhammad Miftahussurur, Ari Fahrial Syam, Langgeng Agung Waskito, Dalla Doohan, Yudith Annisa Ayu Rezkitha, Takashi Matsumoto, Vo Phuoc Tuan, Junko Akada, Hideo Yonezawa, Shigeru Kamiya and Yoshio Yamaoka

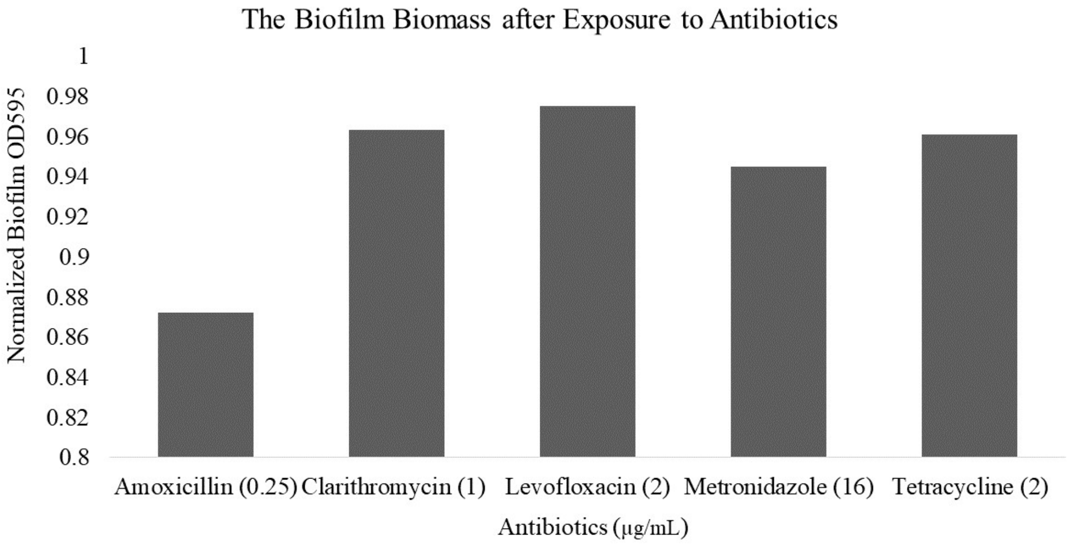

Figure S1. The effect of 24 h antibiotic exposure on the mature biofilm mass of strain MANADO5. The initial OD of the control MANADO5 strain (no antibiotic addition) was 1.72. All OD values were normalized using the OD of this control as 1. The X-axis shows the antibiotic concentration, and the Y-axis shows the optical density of the biofilm, as determined by the crystal violet assay. Exposure to antibiotics for 24 h caused a decrease in the biofilm mass. However, the decrease was not significant.

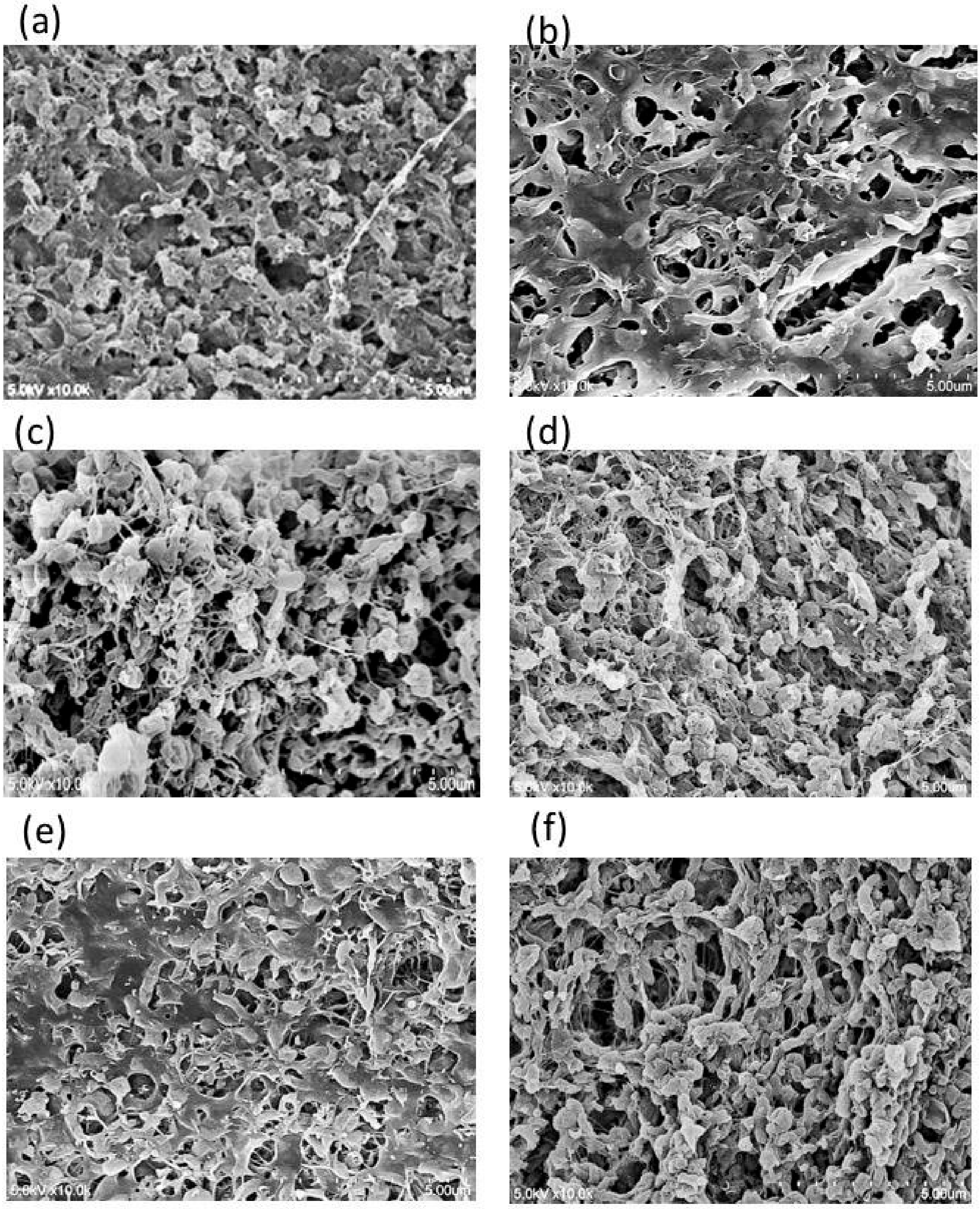

Figure S2. The SEM images of the biofilm of strain MANADO5 24 h after antibiotic exposure at a dose 2 times that of the clinical breakpoint. (**a**) Amoxicillin 1, (**b**) tetracycline 2, (**c**) clarithromycin 1, (**d**) levofloxacin, (**e**) metronidazole, and (**f**) no antibiotics (control).

Table S1. Association of planktonic susceptibility and biofilm formation (total *n* = 101).

Biofilm Formation	*n*	Amoxycillin	Clarithromycin	Levofloxacin	Metronidazole	Tetracycline
			**Mean of MIC (mg/mL)**			
Strong	23	0.016	0.032	0.125	32	0.047
Weak	71	0.016	0.016	0.25	8	0.032
Negative	7	0.016	0.032	0.094	8	0.032
**Total**	**101**	**0.016**	**0.023**	**0.25**	**8**	**0.032**
p		0.39	0.79	0.45	0.43	0.52
**Planktonic Resistance (%)**						
Strong	23	2 (8.7)	2 (8.6)	8 (34.7)	13 (56.5)	0 (0)
Weak	71	2 (2.8)	5 (7.0)	27(38.0)	35 (49.3)	3 (4.2)
Negative	7	0 (0)	1 (14.3)	1(14.3)	2 (28.5)	0 (0.0)
**Total**	**101**	**4 (4)**	**8 (7.9)**	**36 (35.6)**	**50 (49.5)**	**3 (3.0)**
p		0.81	0.41	0.29	0.72	0.42

* *p* value was obtained by the Kruskall-Wallis test of the MIC result among the biofilm groups.

Table S2. The different proportions of resistant *H. pylori* populations obtained by MIC and MBEC measurements.

	MIC Resistant-MBEC Resistant (%)	MIC Resistant-MBEC Sensitive (%)	MIC Sensitive-MBEC Resistant (%)	MIC Sensitive - MBEC Sensitive (%)	*n* isolates
Amoxicillin	1 (9.5)	1 (4.7)	10 (47.6)	9 (42.9)	21
Clarithromycin	2 (9.5)	0 (0.0)	4 (19.0)	15 (71.4)	21
Levofloxacin	9 (42.9)	1 (4.7)	8 (38.1)	3 (14.2)	21
Metronidazole	4 (19.0)	2 (.5)	8 (38.1)	7 (33.3)	21
Tetracycline	0 (0.0)	0.0 (0)	12 (57.1)	9 (42.9)	21
